# Supplementary material for: Zinc Complexes of Fluorosubstituted N-[2-(Phenyliminomethyl)phenyl]-4-methylbenzenesulfamides: Synthesis, Structure, Luminescent Properties, and Biological Activity
Source: Materials (Basel). 2024 Jan 17;17(2):438. doi: 10.3390/ma17020438 (PMC10817688; doi:10.3390/ma17020438)
Supplement: Supplementary file 1 [file materials-17-00438-s001.zip › materials-2677902-supplementary.pdf]

Supplementary Material

Zinc Complexes of Fluorosubstituted *N*-[2-(Phenyliminomethyl)phenyl]-4-methylbenzenesulfamides: Synthesis, Structure, Luminescent Properties, and Biological Activity

Anatolii S. Burlov [1], Valery G. Vlasenko [2], Maxim S. Milutka [1], Yurii V. Koshchienko [1], Vladimir A. Lazarenko [3], Alexander L. Trigub [3], Alexandra A. Kolodina [1], Alexander A. Zubenko [4], Elena V. Braga [5], Alexey N. Gusev [5,*] and Wolfgang Linert [6]

[1] Institute of Physical and Organic Chemistry, Southern Federal University, 344090 Rostov-on-Don, Russia; anatoly.burlov@yandex.ru (A.S.B.); milutka@sfedu.ru (M.S.M.); yukoshch@ipoc.sfedu.ru (Y.V.K.); akolodina@sfedu.ru (A.A.K.)
[2] Institute of Physics, Southern Federal University, 344090 Rostov-on-Don, Russia; v_vlasenko@rambler.ru
[3] National Research Centre "Kurchatov Institute", 123182 Moscow, Russia; lazarenko_va@nrcki.ru (V.A.L.); alexander.trigub@gmail.com (A.L.T.)
[4] North-Caucasian Zonal Scientific Research Veterinary Institute, Branch of the Federal State Budget Scientific Institution "Federal Rostov Agricultural Research Centre", 344006 Rostov-on-Don, Russia; alexsandrzubenko@yandex.ru
[5] General Chemistry Department, Crimean Federal University V.I. Vernadsky, 295007 Simferopol, Russia; braga.yelena@ya.ru
[6] Institute of Applied Physics, Vienna University of Technology, Wiedner Hauptstraße 8-10, 1040 Vienna, Austria; wolfgang.linert@tuwien.ac.at
* Correspondence: galex0330@gmail.com

1. A single-crystal X-ray diffraction

Table S1. Selected crystallographic data for compounds **1d** and **1f**.

**Crystal data**	**1d**	**1f**
CCDC	2299391	2299392
Chemical formula	$C_{20}H_{16}F_2N_2O_2S$	$C_{20}H_{16}F_2N_2O_2S$
M_r	386.41	386.41
Crystal system, space group	Triclinic, $P\bar{1}$	Triclinic, $P\bar{1}$
Temperature (K)	100	100
a, b, c (Å)	8.7350(18), 14.084(3), 17.430(4)	8.8130(18), 10.759(2), 11.274(2)
α, β, γ (°)	101.54(3), 95.00(3), 97.29(3)	98.81(3), 94.35(3), 94.95(3)
V (Å^3)	2070.0 (8)	1048.1 (4)
Z	4	2
F(000)	800	400
D_x (Mg m^{-3})	1.240	1.224

Radiation type	Synchrotron, λ = 0.7454 Å	Synchrotron, λ = 0.7454 Å
No. of reflections for cell measurement	1250	11740
θ range (°) for cell measurement	1.3–29.0	1.9–29.0
μ (mm^{-1})	0.21	0.21
Crystal shape	Block	Block
Colour	Orange	Yellowish orange
Crystal size (mm)	0.2 × 0.1 × 0.1	0.3 × 0.1 × 0.1
**Data collection**		
Diffractometer	Rayonix SX165 CCD	Rayonix SX165 CCD
Radiation source	synchrotron	synchrotron
Scan method	ϕ scans	ϕ scans
Absorption correction	Empirical (using intensity measurements) XDS, Kabsh 2010	Empirical (using intensity measurements) XDS Kabsh
T_{min}, T_{max}	0.945, 1	0.923, 1
No. of measured, independent and observed [$I > 2\sigma(I)$] reflections	35731, 9441, 7652	17934, 4781, 4242
R_{int}	0.045	0.031
θ values (°)	θ_{max} = 29.0, θ_{min} = 1.3	θ_{max} = 29.0, θ_{min} = 1.9
$(\sin \theta/\lambda)_{max}$ ($Å^{-1}$)	0.649	0.649
Range of h, k, l	h = -11 до 11, k = -18 до 18, l = -22 до 22	h = -11 до 11, k = -13 до 13, l = -14 до 14
**Refinement**		
Refinement on	F^2	F^2
$R[F^2 > 2\sigma(F^2)]$, $wR(F^2)$, S	0.050, 0.143, 1.05	0.041, 0.118, 1.07
No. of reflections	9441	4781
No. of parameters	489	248
No. of restraints	0	0

H-atom treatment	H-atom parameters constrained	H atoms treated by a mixture of independent and constrained refinement
Weighting scheme	$w = 1/[s^2(F_o^2) + (0.0752P)^2 + 1.1458P]$ where $P = (F_o^2 + 2F_c^2)/3$	w = 1/[s2(Fo2) + (0.059P)2 + 0.4508P] where P = $(F_o^2 + 2Fc^2)/3$
$(\Delta/\sigma)_{max}$	0.001	0.001
$\Delta\rho_{max}$, $\Delta\rho_{min}$ (e Å^{-3})	0.67, -0.65	0.59, -0.34

Table S2. Selected crystallographic data for compounds **2d, 2h** and **2f**

**Crystal data**	**2h**	**2d**	**2f**
CCDC	2299393	2299394	2299397
Chemical formula	$C_{40}H_{28}F_6N_4O_4S_2Zn·2(CH_4O)$	$C_{40}H_{30}F_4N_4O_4S_2Zn$	$C_{40}H_{30}F_4N_4O_4S_2Zn·0.63(CH_4O)$
M_r	936.24	836.17	856.36
Crystal system, space group	Monoclinic, *C*2/*c*	Monoclinic, *C*2/*c*	Triclinic, $P\bar{1}$
Temperature (K)	100	100	100
a, *b*, *c* (Å)	22.692 (5), 10.840 (2), 16.490 (3)	13.466 (3), 11.878 (2), 22.715 (5)	8.4800 (17), 11.858 (2), 19.371 (4)
α (°)	90	90	81.98 (3)
β (°)	92.35 (3)	102.81 (3)	79.75(3)
γ (°)	90	90	73.71(3)
V (Å^3)	4052.8 (14)	3542.7 (13)	1831.5(7)
Z	4	4	2
F(000)	1920	1712	879
D_x (Mg m^{-3})	1.534	1.568	1.553
Radiation type	Synchrotron, λ = 0.7454 Å	Synchrotron, λ = 0.745 Å	Synchrotron, λ = 0.745 Å
No. of reflections for cell measurement	12500	14600	18350
θ range (°) for cell	2.5–31.0	2.4–31.0	1.1–31.0

measurement			
μ (mm^{-1})	0.89	1.00	0.97
Crystal shape	Block	Block	Block
Colour	Yellow	Yellow	Yellow
Crystal size (mm)	0.1 × 0.05 × 0.05	0.05 × 0.05 × 0.05	0.15 × 0.05 × 0.03
Data collection			
Diffractometer	Rayonix SX165 CCD	Rayonix SX165 CCD	Rayonix SX165 CCD
Radiation source	synchrotron	synchrotron	synchrotron
Scan method	f scans	f scans	f scans
Absorption correction	Empirical (using intensity measurements) XDS, Kabsh 2010	Empirical (using intensity measurements) XDS, Kabsh 2010	Empirical (using intensity measurements) XDS, Kabsh 2010
T_{min}, T_{max}	0.907, 1	0.955, 1	0.866, 1
No. of measured, independent and observed [$I > 2\sigma(I)$] reflections	38650, 5596, 5101	12667, 4881, 3811	30739, 9809, 9043
R_{int}	0.035	0.039	0.071
θ values (°)	θ_{max} = 31.0, θ_{min} = 2.5	θ_{max} = 31.0, θ_{min} = 2.4	θ_{max} = 31.0, θ_{min} = 1.1
$(\sin \theta/\lambda)_{max}$ (Å^{-1})	0.692	0.691	0.691
Range of *h*, *k*, *l*	*h* = -31®31, *k* = -14®14, *l* = -22®22	*h* = -18®18, *k* = -16®16, *l* = -30®31	*h* = -11®10, *k* = -16®16, *l* = -26®26
Refinement			
Refinement on	F^2	F^2	F^2
$R[F^2 > 2\sigma(F^2)]$, $wR(F^2)$, S	0.030, 0.083, 1.04	0.043, 0.107, 1.03	0.043, 0.117, 1.03
No. of	5596	4881	9809

reflections			
No. of parameters	279	250	527
No. of restraints	0	0	4
H-atom treatment	H-atom parameters constrained	H-atom parameters constrained	H-atom parameters constrained
Weighting scheme	$w = 1/[\sigma^2(F_o^2) + (0.0444P)^2 + 3.9985P]$ where $P = (F_o^2 + 2F_c^2)/3$	$w = 1/[\sigma^2(F_o^2) + (0.0405P)^2 + 8.7982P]$ where $P = (F_o^2 + 2F_c^2)/3$	$w = 1/[\sigma^2(F_o^2) + (0.0381P)^2 + 1.4888P]$ where $P = (F_o^2 + 2F_c^2)/3$
$(\Delta/\sigma)_{max}$	0.001	0.001	0.001
$\Delta\rho_{max}$, $\Delta\rho_{min}$ (e, $Å^{-3}$)	0.40, -0.49	0.51, -1.15	0.41, -0.55

2. X-ray absorption

The X-ray Zn K absorption edges of zinc complexes were obtained in the transmission mode at the Structural Materials Science station at the Kurchatov Synchrotron Center (Moscow) [1]. The energy of the electron beam, which was used as a source of X-ray synchrotron radiation, was 2.5 GeV at an average current of 100–120 mA. The X-ray absorption spectra were processed by standard procedures for extracting the background, normalizing to the value of the K-edge jump, and extracting the atomic absorption μ0, after which the Fourier transform of the selected EXAFS (χ) spectrum was performed in the range of photoelectron wave vectors k from 2.5 to 12–13 $Å^{-1}$ with the weight function k^3. The exact values of the nearest environment parameters of the zinc ion in the studied compounds were determined by nonlinear fitting of the parameters of the corresponding coordination spheres when comparing the calculated EXAFS with that extracted by the Fourier filtering method from the full absorption spectrum. This procedure was carried out using the IFFEFIT software package [2]. The phases and amplitudes of photoelectron wave scattering required for constructing the model spectrum were calculated using the FEFF7 program [3]. As the initial atomic coordinates necessary for calculating the scattering phases and amplitudes and further fitting, we used X-ray diffraction data for single crystals of metal complexes with a similar molecular structure from the Cambridge database. The fit quality function Q, which was minimized when finding the structure parameters of the nearest environment, was calculated using the formula:

$$Q^2 = \frac{\sum_{i=1}^{m} w(k_i)\left[k_i\chi_{\exp}(k_i) - k_i\chi_{th}(k_i)\right]^2}{\sum_{i=1}^{m} w(k_i)\left[k_i\chi_{\exp}(k_i)\right]^2}$$

where $w(k_i)$ is a weighting function, m is the number of experimental points, $\chi_{data}(R_i)$ and $\chi_{th}(R_i)$ are the EXAFS functions in the R-space.

3. Biological activity

Antibacterial activity was studied by the agar diffusion technique as described in [4,5]. We used discs with a diameter of 6 mm, made of filter cardboard to determine the sensitivity to antimicrobial preparations (ND-PMP-1 produced by the Saint-Petersburg Pasteur Institute). At the first stage (screening of active compounds), two cultures were used: gram-negative bacteria *Escherichia coli F 50* and gram-positive bacteria *Staphylococcus aureus 6538 P*. Solutions of the tested compounds were applied to the discs. The loading of each compound was 15 μg per disk. The discs were placed on the lawn of a bacterial culture seeded on nutrient agar in Petri dishes by washing a daily bacterial culture with a density of 5 units according to the optical standard. The activity of the compounds was assessed by the size of the zone of inhibition of the growth of the bacterial culture. The reference preparations were furazolidone and ciprofloxacin.

The study of the fungistatic activity of new substances was carried out on the culture of fungi of the genus *Penicillium*, species *Penicillium italicum Wehmer* (1894) (field isolate, from the collection of micromycetes of the laboratory of mycotoxicology of the North-Caucasian Zonal Scientific Research Veterinary Institute, Russia) according to the method [4]. A commercial fungicide fundazol was used for comparison. An aqueous solution of the test compounds or fundazol for comparison was placed on a disc of filter paper (ND-PMP-1 produced by the Saint-Petersburg Pasteur Institute) in an amount of 15 μg per disc with a diameter of 6 mm.

The antiprotozoal activity was studied against infusoria *Colpoda steinii* (field isolate from the collection of the laboratory of parasitology of the North-Caucasian Zonal Scientific-Research Veterinary Institute, Russia) using a method of serial dilutions, which was described elsewhere [4-6]. Reference preparations were: Baycox - 2.5% solution of toltrazuril and chloroquine in the form of aqueous solutions at the same concentrations as the test compounds.

References

1. Chernyshov, A.A.; Veligzhanin, A.A.; Zubavichus, Ya.V. Structural Materials Science End-Station at the Kurchatov Synchrotron Radiation Source: Recent Instrumentation Upgrades and Experimental Results. *Nucl. Instr. Meth. Phys. Res. (A)*, **2009**, *603*, 95-98. [doi 10.1016/j.nima.200812.167]
2. Newville, M. EXAFS analysis using FEFF and FEFFIT. *J. Synchrotron Rad*. **2001**, *8*, 96-100. [doi 10.1107/S0909049500016290]
3. Zabinski, S.I.; Rehr, J.J.; Ankudinov, A.; Alber, R.C. Multiple-scattering calculations of x-ray-absorption spectra. *Phys. Rev*. **1995**, *52*, 2995-11. [doi 10.1103/PhysRevB.52.2995]
4. Burlov, **A**.S.; Vlasenko, V.G.; Koshchienko, Yu.V.; Makarova, N.I.; Zubenko, A.A.; Drobin, Yu.D.; Fetisov, L.N.; Kolodina, A.A.; Zubavichus, Ya.V.; Trigub, A.L.; Levchenkov, S.I.; Garnovskii, D.A. Synthesis, characterization, luminescent properties and biological activities of zinc complexes with bidentate azomethine Schiff-base ligands. *Polyhedron,* **2018**, *154*, 65–76. [https://doi.org/10.1016/j.poly.2018.07.034]
5. Burlov, A.S.; Vlasenko, V.G.; Koshchienko, Yu.V.; Makarova, N.I.; Zubenko, A.A.; Drobin, Yu.D.; Borodkin, G.S.; Metelitsa, A.V.; Zubavichus, Ya.V.; Garnovskii, D.A. Complexes of zinc(II) with N-[2-(hydroxyalkyliminomethyl)phenyl]-4-methylbenzenesulfonamides: synthesis, structure, photoluminescence properties and biological activity. *Polyhedron* **2018**, 144, 249-258. [doi.org/10.1016/j.poly.2018.01.020]
6. Fetisov, L.N.; Zubenko, A.A.; Bodryakov, A.N.; Bodryakova, M.A. Search for new protistocidal drugs. Proc. Int. Parasitol. Symp. “Modern Problems of General and Partial Parasitology”, **2012**, 70.
